# Supplementary material for: Regulation of Ion Permeation of the KcsA Channel by Applied Midinfrared Field
Source: Int J Mol Sci. 2022 Dec 29;24(1):556. doi: 10.3390/ijms24010556 (PMC9820211; doi:10.3390/ijms24010556)
Supplement: Supplementary file 1 [file ijms-24-00556-s001.zip › ijms-2065556-supplementary.pdf]

Supporting Information

Contents

1. Simulation system preparation
2. Simulation methods
3. Setting of charge difference parameters
4. High-frequency electromagnetic fields act on the KcsA channel
5. Ion flux under different electric field strength
6. Vibration spectrum of -C=O group
7. Stability of the SF regional structure

References

1. Simulation system preparation

The simulated systems selected the KcsA (PDB:5VK6) channel which has an open inner helix bundle gate and an activated selectivity filter in Fig.1. This channel was embedded in a patch of a palmitoyl-oleoyl-phosphatidylcholine (POPC) membrane containing 134 lipid molecules, which was then hydrated by 10359 TIP3P water molecules. KCl (0.3M) was also added to neutralize the system, resulting in a simulation system of 80.2×80.2×94.4 $Å^3$.

All molecular dynamics (MD) simulations were performed using GROMACS software version 2019.3 with CHARMM36 force field. Periodic boundary conditions were applied in the XYZ direction. PME was used to treat electrostatic interactions exceeding the 1nm cutoff, and set the cutoff of vdW interactions to 1nm.The LINCS constraint algorithm was used to reset bonds after an unconstrained update of 2 fs. The pressure and temperature were held at 1 atm and 300 K by the semi-isotropic Parrinello-Rahman barostat and the v-rescale thermostat respectively. The systems were performed a steepest descent energy minimization and then equilibrated until the system stabilizes. In the 5 ns of the NVT and 15 ns of NPT equilibration simulations, the heavy atoms of protein were restrained with a force constant of 1000 kJ mol^{-1} nm^{-2} to their starting positions. Lipids, ions, and water were allowed to move freely during equilibration.

2. Simulation methods

2.1 The ion imbalance method

The Computational Electrophysiology (CompEL) method in GROMACS is used to simulate the ion flux through KcsA channel[1], driven by transmembrane potentials (TM). We duplicated the well-equilibrated single-layered system in the direction of the membrane normal (Z-axis) to prepare a double-layered system for CompEL simulations. The transmembrane potential is caused by an ion imbalance between the two sides of the membrane, which is also called the ion imbalance (IIMB) method[1,2]. The .mdp file was set to force the small charge imbalance Δq and we set the ion imbalance between the compartments as 4e.

2.2 The combined method

At present, there are mainly two methods to construct transmembrane potential in molecular simulation, one is the IIMB method mentioned above, the other is the constant electric field (CEF) method[3-5]. In the CEF method, the (TM) potential is simulated by adding an applied electric field along the membrane normal direction. The TM voltage ΔV can be calculated by $\Delta V = \vec{E} \cdot L$, where L is the length of the simulation box, $\vec{E}$ is the strength of the electric field, and the charged particles are

subjected to a constant additional force $\vec{F} = q_i\vec{E}$.

In the combined model, the TM potential is constructed by the IIMB method, and when the number of potassium (K) ions on each side of the membrane deviates from the reference count, the forced exchange between K^+ ions and water was carried out, which was similar to the ion pump in real cells. The CEF method is used to apply an applied electric field to the whole simulation system, so as to simulate the influence of the THz electric field on KcsA K^+ ion channel.

The simulation results of the IIMB method were used as the reference for the simulation without applied electric field, and the CEF-IIMB combined method is used for the simulation of midinfrared (MIR) electric field with different frequencies acting on KcsA channel.

3. Setting of the charge difference parameter

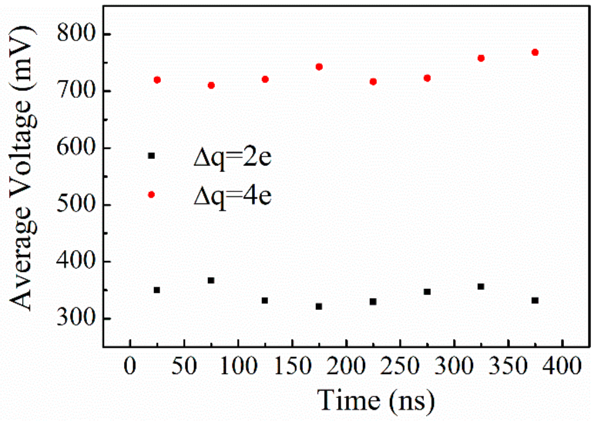

Figure S1 | Membrane voltage at different charge differences.

The transmembrane potential in CompEL is achieved by sustaining a small imbalance of charges Δq across the membrane. Fig.S1 shows the average voltage of several independent simulation results every 50ns when the simulation system was set with different charge differences. When Δq=2e, the voltage fluctuates around 330mV, and when Δq=4e, the voltage fluctuates around 720mV. Fig.S1 shows the average voltage of several independent simulation results every 50ns when the simulation system is set with different charge differences. When Δq=2e, the voltage fluctuates around 330mV, and when Δq=4e, the voltage fluctuates around 720mV. Fig. S2 shows the ion flux at different charge differences. The average ion flux curve of Δq=2e is obtained by independently simulating 20 groups with a total time of 8μs, but the ion flux curve has not yet converged. The transmembrane voltage when Δq=4e is relatively high, but compared to the case of Δq=2e, there is a more stable ion current. At the same time, the effect of the external MIR electric field on the ion flux can be seen more clearly.

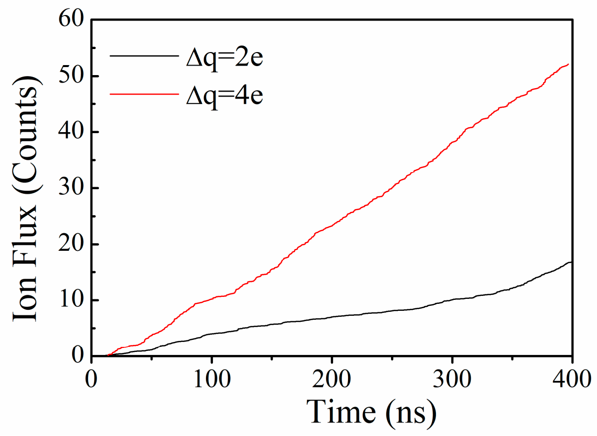

Figure S2 | Ion flux at different charge differences.

4. High frequency electromagnetic fields act on KcsA channels

The wavelength of 1THz electromagnetic wave in free space is

$$\lambda = \frac{c}{f} = \frac{3 \times 10^8}{1 \times 10^{12}} = 3 \times 10^{-4} m = 300 \mu m$$

and the size of the single box we simulated is 80.2×80.2×94.4 $Å^3$. As the wave of terahertz electromagnetic wave is longer than the size of the simulated region, quasi-stable approximation is adopted in the simulation process.

For the plane electromagnetic wave with electric field intensity $E(t)$ and magnetic field intensity $H(t)$ in free space, the electric field force f_e and magnetic field force f_m subjected to charged particles with charge q and velocity v are respectively:

$$f_e = qE(t)$$

$$f_m = qv\mu_0 H(t)$$

Then the ratio of the magnetic field force to the electric field force on the ion is:

$$\frac{f_m}{f_e} = v\mu_0 \frac{H(t)}{E(t)} = v\mu_0 \sqrt{\frac{\varepsilon_0}{\mu_0}} = v\sqrt{\mu_0 \varepsilon_0} = \frac{v}{c}$$

Because charged particles travel much less than the speed of light, the electric field force on the charged particles is much greater than the magnetic field force. Therefore, in the simulation, the influence of the high frequency magnetic field can be ignored and only the influence of the high frequency electric field can be considered. Therefore, for the external sinusoidal alternating electric field with amplitude of electric field intensity E_0 and frequency $2\pi\omega$ Hertz, the following expression can be adopted:

$$E(t) = E_0 \cos\left[\omega(t - t_0)\right]$$

5. Ion flux under different electric field strength

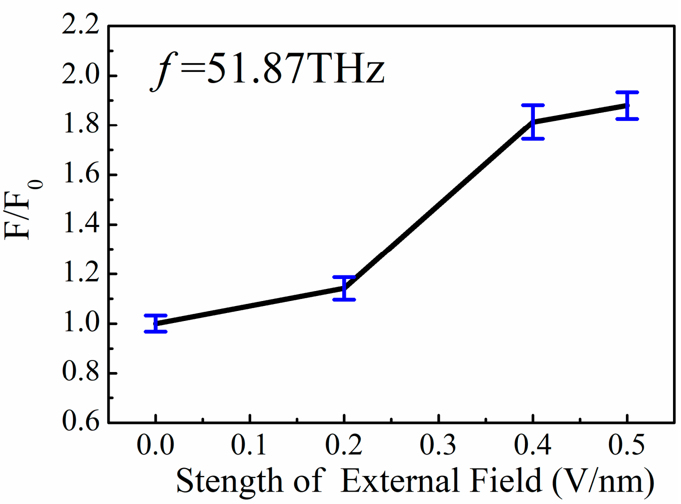

Figure S3 | The influence of electric field amplitude on ion flux. The ion flux is normalized the situation without external field (F_0).

We simulated the dynamics for the four cases where the frequency of the external electric field was 51.87 THz and the amplitude was 0.2V/nm, 0.4V/nm, 0.5V/nm and 0.6V/nm, respectively. Similar to the simulation process without MIR electric field, we performed 8 independent simulations with different amplitudes of the 51.87 THz electric field for a total time of 3.2μs, and averaged the ion fluxes obtained from different amplitudes simulation. The ion fluxes under electric fields with different amplitudes were normalized to the ion flux without the external electric field, shown in Fig. S3. The ion flux with an amplitude of 0.2V/nm is close to the ion flux without MIR field, which is 1.14 times that without MIR field, while the ion flux with an amplitude of 0.4V/nm is 1.8 times that without MIR field. The ion flux at a value of 0.5V/nm is close to the ion flux at 0.4V/nm, which is 1.87 times that of without the external MIR field. We have also simulated the case where the amplitude is 0.6V/nm, but it may be because the field strength exceeds the threshold, the simulation system always collapsed around 200ns. So we finally chose 0.4V/nm as the amplitude of the external electric field when comparing the influence of frequency on ion flux.

6. Vibration spectrum of -C=O group

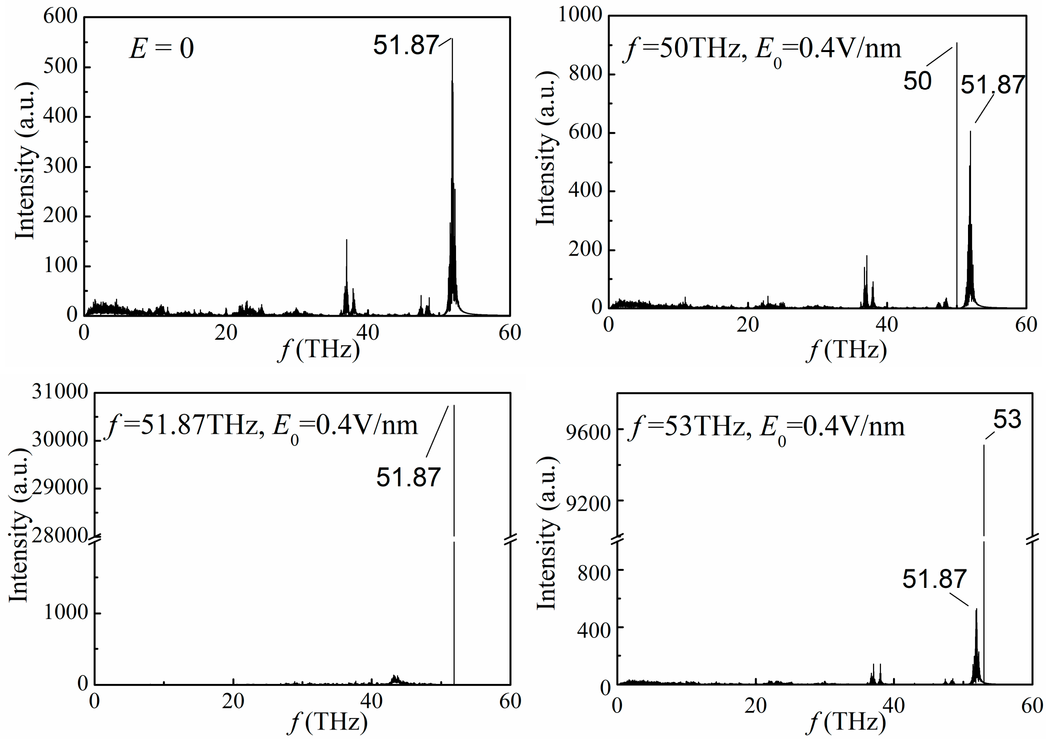

Figure S4 | Vibration spectrum of the -C=O group in the SF region with MIR electric fields of different frequencies. When the frequency of applied electric field is 51.87 THz, the -C=O group has strong absorption to the applied electric field.

7. Stability of the SF regional structure

Table S1. The average distance between the oxygen atoms of the symmetric Y78 residues

Independent simulations	E=0	50THz	51.87THz	53THz
1	0.98759	0.66758	0.51405	1.09076
2	0.52008	1.05897	0.51658	0.82479
3	0.48367	0.48762	0.51076	0.74636
4	0.52972	0.94644	0.51418	0.50848
5	0.50037	0.57805	0.50874	0.50645
6	0.59966	0.5571	0.51335	0.85751
7	0.51874	0.78586	0.51294	0.74143
8	0.49245	0.50008	0.51294	0.53196
Variance	2.49581E-02	3.97921E-02	4.82247E-06	3.67407E-02

(Unit: nm)

The initial distance between the oxygen atoms of the symmetric Y78 residues in the SF is 0.54 nm. When the frequency of applied electric field is 51.87 THz, the fluctuation value of independently repeated simulation for 8 times is the smallest, and fluctuates around 0.54 nm. The fluctuation values of the case of 50THz field and 53THz are very large.

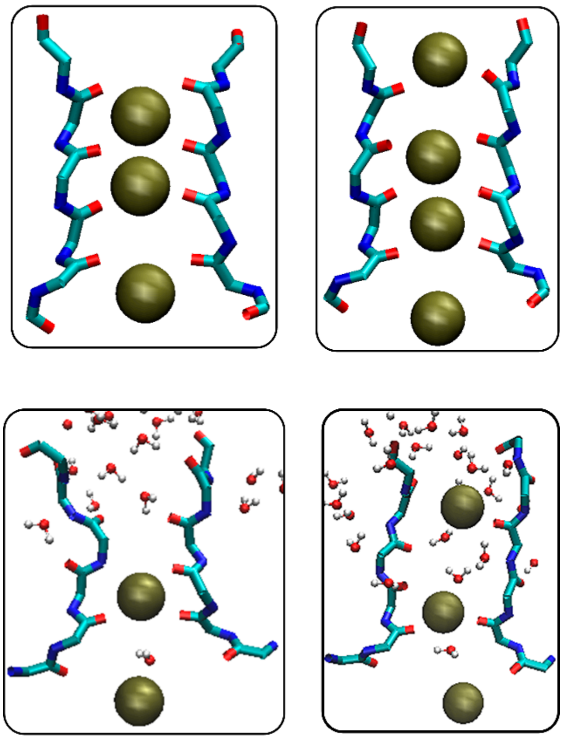

Figure S5 | The graph above is a random screenshot during the simulation with the applied electric field of 51.87 THz. The plot below is a random screenshot during a simulation with the applied electric field of 53 THz. The structure of the SF with the field at 51.87 THz is significantly more stable.

References

(1) Kutzner, C.; Grubmüller, H.; de Groot, B. L.; Zachariae, U. Computational Electrophysiology: The Molecular Dynamics of Ion Channel Permeation and Selectivity in Atomistic Detail. Biophys. J. 2011, 101, 809−817.

(2) Sachs, J. N., P. S. Crozier, and T. B.Woolf. Atomistic Simulations of Biologically Realistic Transmembrane Potential Gradients. J. Chem. Phys. 2004, 121, 10847–10851.

(3) Roux, B. The Membrane Potential and its Representation by a Constant Electric Field in Computer Simulations. Biophys. J. 2008, 95, 4205-4216.

(4) Delemotte, L.; Dehez, F.; Treptow, W.; Tarek, M. J. Modeling Membranes under a Transmembrane Potential. Phys. Chem. B. 2008, 112, 5547−5550.

(5) Gumbart, J.; Khalili-Araghi, F.; Sotomayor, M.; Roux, B. Con-stant Electric Field Simulations of the Membrane Potential Il-lustrated with Simple Systems. Biochim. Biophys. Acta, Biomembr. 2012, 1818, 294−302.
